# Risk factors for COPD exacerbations and mortality, and variation between primary care settings: the PRAXIS cohort study in Sweden

**DOI:** 10.1136/fmch-2025-003713

**Published:** 2026-02-19

**Authors:** Carolina Smith, Ayako Hiyoshi, Tor Arnison, Gabriella Eliason, Maaike Giezeman, Mikael Hasselgren, Christer Janson, Mikael Karlsson, Marta A Kisiel, Karin Lisspers, Anna Nager, Hanna Sandelowsky, Björn Ställberg, Josefin Sundh, Scott Montgomery

**Affiliations:** 1 Clinical Epidemiology and Biostatistics, School of Medical Sciences, Faculty of Medicine and Health, Örebro University, Örebro, Sweden; 2 Centre for Clinical Research and Education, Region Värmland, Karlstad, Sweden; 3 Department of Public Health Sciences, Stockholm University, Stockholm, Sweden; 4 Department of Respiratory Medicine, Faculty of Medicine and Health, Örebro University, Örebro, Sweden; 5 School of Medical Sciences, Faculty of Medicine and Health, Örebro University, Örebro, Sweden; 6 Department of Medical Sciences, Respiratory, Allergy and Sleep Research, Uppsala University, Uppsala, Sweden; 7 Occupational and Environmental Medicine, Uppsala University, Uppsala, Sweden; 8 Department of Public Health and Caring Sciences, Uppsala University, Uppsala, Sweden; 9 Division of Family Medicine and Primary Care, Karolinska Institutet, Stockholm, Sweden; 10 Academic Primary Health Care Centre, Region Stockholm, Stockholm, Sweden; 11 Clinical Epidemiology Division, Department of Medicine, Karolinska Institutet, Stockholm, Sweden

**Keywords:** Respiratory System, Chronic Disease, Primary Health Care, Epidemiology

## Abstract

**Objective:**

We aimed to examine the variability of chronic obstructive pulmonary disease (COPD) exacerbations and mortality, between primary healthcare centres, and their associations with comorbid diseases and body mass index, during an 8-year follow-up.

**Design:**

This was a cohort study using multilevel modelling with follow-up from 2014 to 2022. Data came from questionnaires in 2014 and 2022 and medical record reviews between 2004 and 2014. The main outcomes were exacerbations in 2022 and mortality by 2022. Exacerbations were defined as any emergency visit, and/or use of oral steroids or antibiotics due to worsening of COPD symptoms during the previous 6 months.

**Setting:**

The PRAXIS study included patients at 76 primary healthcare centres in central Sweden.

**Participants:**

Primary care patients aged ≤75 years and with a diagnosis of COPD in their medical records between 2007 and 2010 were included in 2014 (n=1163) and followed up in 2022 (n=906). There were no other exclusion criteria.

**Results:**

The 809 patients with complete data attended 70 primary care centres. Multilevel multinomial regression estimated risks of exacerbations and mortality, calculating relative risk ratios (RRRs) with 95% CIs. The intraclass correlation coefficient (ICC) quantified the proportion of variance attributed to variability between centres. The ICC was 0.024, indicating 2.4% of the variation was explained by differences between centres. Patients with a history of depression in 2014 had an increased risk of subsequent exacerbations (RRR 1.95, 95% CI 1.13 to 3.39). For mortality, there were associations with history of anxiety, RRR 3.71 (95% CI 2.06 to 6.87), or cardiovascular disease, especially chronic heart failure, RRR 2.69 (95% CI 1.36 to 5.33). Body mass index had a U-shaped association with mortality.

**Conclusions:**

The variability between centres was small and patient factors appear to be of more importance for COPD exacerbations and mortality than differences between these primary care settings. As expected, pre-existing cardiovascular disease is associated with future excess mortality risk, but, notably, anxiety may also be an important risk factor. Individualised care and management of comorbidity is thus essential among patients in primary care with COPD.

**WHAT IS ALREADY KNOWN ON THIS TOPIC**

- Comorbidity is common among patients with chronic obstructive pulmonary disease (COPD) in primary care and affects mortality and risk of exacerbations. It is not clear how differences between healthcare centres (eg, number of patients, location and staffing) affect these COPD outcomes.

**WHAT THIS STUDY ADDS**

- This cohort study of patients with COPD with an 8-year follow-up suggests that individual patient factors are of more importance for COPD outcomes than differences between these healthcare centres in Sweden.

**HOW THIS STUDY MIGHT AFFECT RESEARCH, PRACTICE OR POLICY**

- Individualised management of patients with COPD with comorbid diseases is required to identify patients who would benefit from more comprehensive monitoring or preventative interventions, possibly including those with higher levels of anxiety.

## Introduction

 Patients with chronic obstructive pulmonary disease (COPD) and comorbidity face increased risks of exacerbations, mortality and poor quality of life.^1^ On average, a patient with COPD has five other chronic diseases.^2^ For example, depression and cancer have been associated with frequent exacerbations and increased healthcare costs.^3 4^ Anxiety, cancer and cardiovascular disease are associated with a higher risk of mortality.^5^ Body mass index (BMI) is also associated with COPD outcomes. For mortality, the relationship with BMI is U-shaped, with higher risk for those with low or high BMI.^6^ For exacerbations, low BMI has been associated with increased risk.^7^

In Sweden, most patients with COPD, even those with severe COPD and comorbidity, are mainly managed in primary care.^8^ Primary care centres may have differences in location (rural or urban, with variation in socioeconomic characteristics of patients), number of patients, use of locum physicians and access to specialist primary care nurses for assessment and management of patients with COPD (including nurse-led COPD clinics within the healthcare centre). Previous research has found that only a small part of the variation in health outcomes can be attributed to differences in staffing or other aspects of healthcare centres in countries outside of Scandinavia.^9^ The degree of variation between primary healthcare centres in Sweden for COPD outcomes is unclear.

This study aims to examine the associations of comorbid diseases and BMI with subsequent exacerbations and mortality in patients with COPD, and specifically to assess the variation of these associations between healthcare centres using multilevel modelling, during an 8-year follow-up.

## Methods

### Study population and design

**Figure 1:**
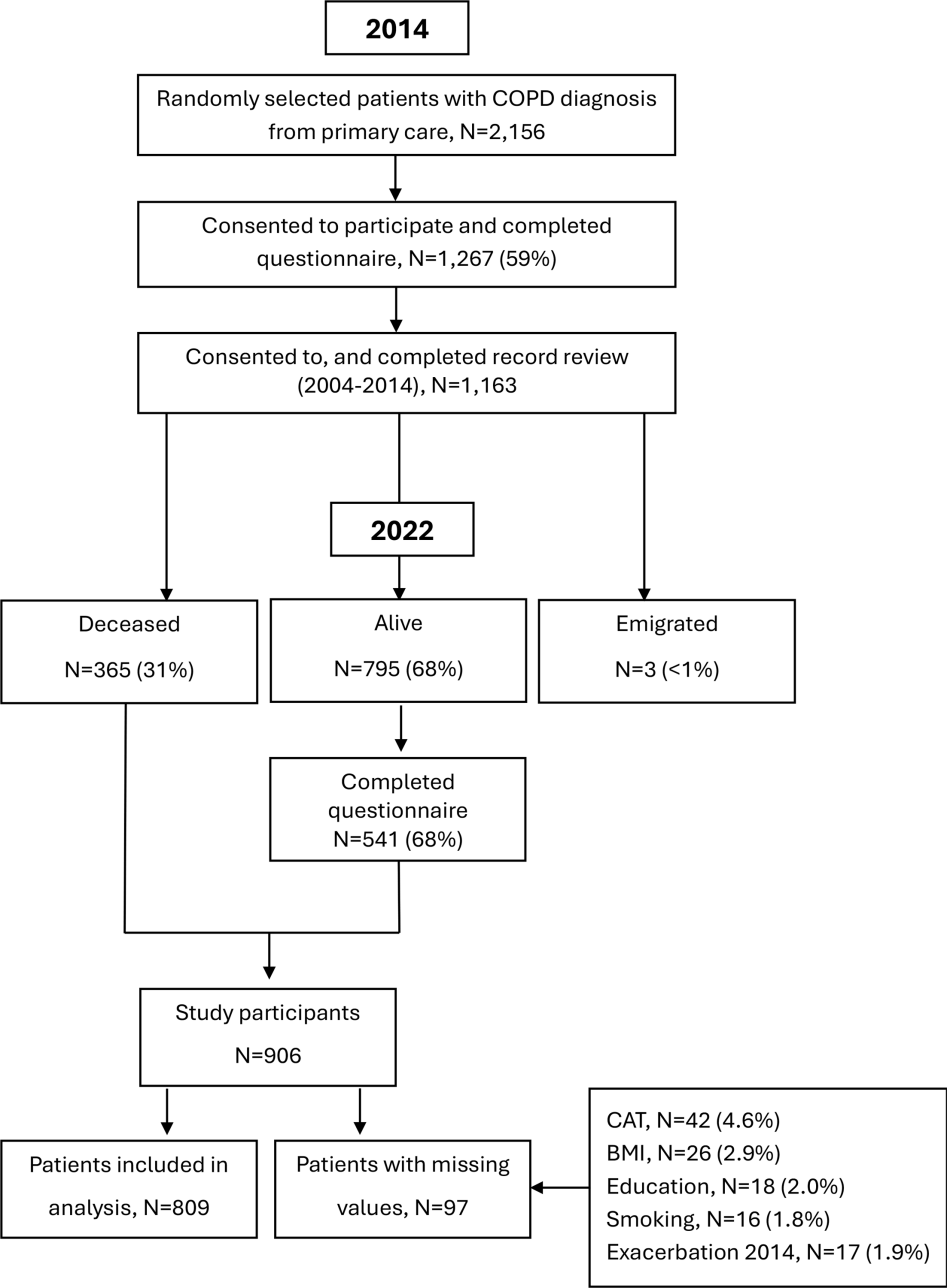
Flow chart of the study population. BMI body mass index; CAT, COPD Assessment Test; COPD, chronic obstructive pulmonary disease.

The study population consisted of the second PRAXIS COPD cohort (https://praxisstudien.com/). It was created in 2014, by inviting randomly selected patients who were treated at 76 primary healthcare centres in central Sweden with a physician’s diagnosis of COPD (ICD-10 code J44) in their medical records between 2007 and 2010, and aged ≤75 years. There were no other exclusion criteria. Among 2156 patients who were invited to the study, 1267 (59%) consented and returned a self-completion questionnaire in 2014 (figure 1). Some 1163 patients agreed to a medical record review (2004–2014) performed by two research nurses using a detailed template. In 2022, the 795 (68%) patients who were alive were invited to participate in a follow-up, and 541 (68%) completed the second questionnaire. The participating healthcare centres filled in a questionnaire in 2012 about the characteristics of their centre.

This was a cohort study using multilevel modelling with follow-up from 2014 to 2022. Individuals with missing data for covariates (N=97) were excluded from the analysis; for details, see figure 1.

### Setting and exposures

#### Primary healthcare centres

The healthcare centres were in seven county councils in central Sweden, where the largest city has approximately 250 000 inhabitants. Both private and state-operated practices were included. Patients were assigned to the centre where they were listed at study start in 2014. Data on centre characteristics such as number of patients, presence of nurse-led COPD clinic and guidelines for COPD were obtained from the healthcare centre questionnaire in 2012.

#### Comorbidity

Comorbid diseases between 2004 and 2014 were identified in the medical record review by the ICD-10 codes or as free text. Type 1 and 2 diabetes were recorded together as diabetes.

#### Body mass index

Self-reported weight and height were obtained from the questionnaire in 2014. BMI was calculated as the ratio between weight in kilograms (kg) and height in metres squared (m^2^).

### Outcomes

Information on exacerbations during the previous 6 months was obtained from the 2022 questionnaire and defined as any unscheduled/emergency visit to primary or secondary care, and/or use of oral steroids, and/or antibiotics due to worsening of COPD symptoms. Data on mortality were supplied by Statistics Sweden. As the mortality rate was relatively high in the study population, mortality was included as an outcome to consider competing risks, rather than excluding those who had died. Therefore, the outcome variable had three categories: alive without any reported exacerbation during the previous 6 months at follow-up in 2022, alive with at least one reported exacerbation during the previous 6 months at follow-up in 2022 or died by 2022.

### Covariates

COPD exacerbations in 2014 were defined in the same way as described for 2022. Data were obtained from the 2014 questionnaire. To measure COPD-specific health status, we used the COPD Assessment Test (CAT),^10^ which includes eight items on symptoms scored by the patient on a scale from 0 to 5: the sum may vary between 0 and 40. A higher score indicates worse health status. We used the CAT score at baseline in 2014 as a measure of pre-existing COPD severity.

Data on smoking and attained level of education were obtained from the 2014 questionnaire. Smoking was categorised as never smoker, former smoker or current smoker (daily or occasionally). Education, as a marker of socioeconomic characteristics, was dichotomised to identify at least 2 years of continued education beyond the 9 years of Swedish compulsory school or less.

### Statistical analyses

Categorical variables were described using frequencies and percentages, while continuous variables were described using means and SD or medians and 25–75 IQR. The χ² test and Student’s t-test were used to compare differences between the participants and those lost to follow-up.

We used multilevel multinomial logistic regression to assess the risk of exacerbations and mortality, including the variation between the primary healthcare centres. To evaluate variation in outcomes across centres, we fitted three models: (1) an empty model without covariates, (2) a model including all comorbid diseases and individual characteristics and (3) a model additionally including centre characteristics. The variance from each model was used to calculate the intraclass correlation coefficient (ICC) which quantifies the proportion of variance that is attributed to the variability between the healthcare centres.^11^ ICC was calculated as: variance/(variance+(π^2^/3)). The multinomial component of the model compared the likelihood of the two outcomes, alive with at least one reported exacerbation in 2022 or died by 2022, against the reference category of being alive with no reported exacerbations in 2022. We estimated relative risk ratios (RRRs) with 95% CIs. The comorbid diseases were analysed separately without adjustment, and in the main analysis, all comorbid diagnoses were included together with sex, age, smoking, attained level of education, BMI, CAT score and exacerbations in 2014. Chronic heart failure, ischaemic heart disease and atrial fibrillation were included as separate exposure variables in the main analysis. In a second analysis, they were combined into one variable: cardiac disease. Similarly, anxiety and depression were analysed separately in the main analysis, and as a combined variable (depression or anxiety) in a second analysis. Additional analyses were stratified by sex, and differences assessed by multiplicative interaction testing.

BMI was modelled as a continuous variable with restricted cubic splines with three knots positioned at the 10th, 50th and 90th percentiles, to allow for a non-linear relationship with the outcomes and to provide comprehensive adjustment, as use of a categorical variable may lead to inaccurate estimations.^12^ Age was modelled using age and age squared. The CAT score was modelled as a continuous variable.

### Sensitivity analyses 

We performed three sensitivity analyses. First, to examine the effect of missing data, we compared RRRs obtained from unadjusted analysis including and excluding individuals with missing data from one or more covariates. Second, since the main analysis included deceased individuals, we performed multilevel logistic regression analysis to assess the risk of exacerbation only among those who remained alive. Third, small group sizes (<5 patients per centre) may have a disproportionate influence on the estimates, so we performed an analysis excluding individuals who belonged to centres with fewer than five individuals.^13^

Data management and statistical analysis were performed using Stata V.18.

### Patient and public involvement

There was no specific patient or public involvement in planning or execution of the study.

## Results

### Population and healthcare centre characteristics

At study start in 2014, 59% of the invited patients completed the questionnaire. The patients who did not respond tended to be younger, with a mean age of 65.4 years compared with 67.5 years among respondents (p<0.001). At follow-up in 2022, 68% of the survivors completed the new questionnaire. While there were no statistically significant differences in age, sex, education, BMI or number of comorbid diseases between the participating patients and the non-participants, the participating patients had a slightly lower mean CAT score in 2014 than the non-participants: 14 compared with 15, respectively (p=0.04).

The final study population consisted of 809 patients with COPD, attending 70 primary healthcare centres. The centres had between 2000 and 20 300 registered patients (median 9605, IQR 6612–12 400). The majority of centres, 60 of 70 (86%), had nurse-led COPD clinics and 61 (87%) had local or regional guidelines for management of COPD. The median number of participating patients per healthcare centre was 12, IQR 10–14 and range 1–18. There were five centres with fewer than five participants. The patients’ median age in 2014 was 69 (IQR 64–72), and 53.5% were female (see table 1).

**Table 1:** Baseline characteristics in 2014

	No exacerbation in 2022	Exacerbations in 2022	Deceased by 2022
N (%)	355 (43.9)	140 (17.3)	314 (38.8)
Sex, n (%)			
Female	197 (55.5)	82 (58.6)	154 (49.0)
Male	158 (44.5)	58 (41.4)	160 (51.0)
Age, n (%)			
<60 years	51 (14.4)	28 (20.0)	19 (6.1)
60–64 years	63 (17.8)	17 (12.1)	30 (9.6)
65–69 years	108 (30.4)	37 (26.4)	89 (28.3)
70–74 years	105 (29.6)	44 (31.4)	120 (38.2)
≥75 years	28 (7.9)	14 (10.0)	56 (17.8)
Age (years), median (IQR)	68 (63–71)	69 (62–72)	70 (67–73)
BMI, n (%)			
<18.5 kg/m^2^	5 (1.4)	3 (2.1)	23 (7.3)
18.5–24.9 kg/m^2^	112 (31.6)	55 (39.3)	136 (43.3)
25–29.9 kg/m^2^	154 (43.4)	48 (34.3)	89 (28.3)
≥30 kg/m^2^	84 (23.7)	34 (24.3)	66 (21.0)
CAT score, mean (SD)	12.6 (7.5)	17.2 (7.8)	16.9 (8.5)
Smoking, n (%)			
Never smoker	15 (4.2)	7 (5.0)	11 (3.5)
Former smoker	229 (64.5)	87 (62.1)	197 (62.7)
Current smoker	111 (31.3)	46 (32.9)	106 (33.8)
Education, n (%)			
Low	209 (58.9)	76 (54.3)	196 (62.4)
High	146 (41.1)	64 (45.7)	118 (37.6)
Exacerbation in 2014, n (%)	70 (19.7)	66 (47.1)	120 (38.2)
Comorbidity, n (%)			
Chronic heart failure	19 (5.4)	10 (7.1)	53 (16.9)
Ischaemic heart disease	40 (11.3)	23 (16.4)	64 (20.4)
Atrial fibrillation	19 (5.4)	12 (8.6)	41 (13.1)
Stroke/TIA	27 (7.6)	9 (6.4)	35 (11.2)
Hypertension	193 (54.4)	69 (49.3)	176 (56.1)
Depression	61 (17.2)	39 (27.9)	52 (16.6)
Anxiety	38 (10.7)	17 (12.1)	57 (18.2)
Diabetes	55 (15.5)	24 (17.1)	55 (17.5)
Osteoporosis	22 (6.2)	16 (11.4)	35 (11.2)
Cancer	26 (7.3)	20 (14.3)	52 (16.6)
Combined measures, n (%)			
Cardiac disease*	68 (19.2)	32 (22.9)	103 (32.8)
Depression/anxiety†	74 (20.9)	40 (28.6)	83 (26.4)

* Combined variable for chronic heart failure, ischaemic heart disease and atrial fibrillation.

† Combined variable for depression and anxiety.

BMI, body mass index; CAT, COPD assessment test; COPD, chronic obstructive pulmonary disease; TIA, transient ischaemic attack.

### Variation by healthcare centres

The variance in the unconditional (empty) model was 0.04 (95% CI 0.01 to 1.20), with ICC 0.012. When comorbidity and other patient characteristics were added to the model, the variance was 0.08 (95% CI 0.01 to 0.73), giving an ICC of 0.024. When centre characteristics (existence of nurse-led COPD clinic) were added, the variance was 0.06 (95% CI 0.01 to 1.13), ICC 0.018. This indicates that only 1.2%–2.4% of the risk of exacerbations or mortality is explained by differences between healthcare centres and, thus, the variability in outcomes between centres was small.

### Exacerbations and mortality

The adjusted RRRs were obtained by mutually adjusting for all comorbid diseases as well as age, sex, smoking, attained level of education, BMI, CAT score and exacerbations in 2014 (see table 2). Patients with a history of depression had a statistically significantly increased risk ratio for having at least one exacerbation during the previous 6 months in 2022, compared with having no recent exacerbations, RRR 1.95 (95% CI 1.13 to 3.39). Exacerbations in 2014 were also associated with an increased risk of subsequent exacerbations, RRR 2.74 (95% CI 1.71 to 4.39). We did not find any statistically significant associations between cardiac disease, diabetes, smoking or education and risk of exacerbations.

**Table 2:** Multilevel analysis of COPD outcomes: exacerbations and mortality

N=809	Exacerbations in 2022, n=140		Deceased by 2022, n=314	
	Unadjusted	Adjusted	Unadjusted	Adjusted
	RRR (95% CI)	RRR (95% CI)	RRR (95% CI)	RRR (95% CI)
Comorbidity				
Chronic heart failure	1.36 (0.61 to 3.01)	0.88 (0.34 to 2.25)	3.59 (2.07 to 6.22)	2.69 (1.36 to 5.33)
Ischaemic heart disease	1.57 (0.90 to 2.74)	1.33 (0.71 to 2.47)	2.04 (1.32 to 3.15)	1.30 (0.78 to 2.17)
Atrial fibrillation	1.65 (0.78 to 3.51)	1.95 (0.81 to 4.73)	2.65 (1.50 to 4.68)	1.41 (0.70 to 2.84)
Stroke/TIA	0.84 (0.38 to 1.85)	0.79 (0.34 to 1.84)	1.54 (0.90 to 2.62)	1.52 (0.80 to 2.87)
Hypertension	0.82 (0.55 to 1.22)	0.77 (0.48 to 1.23)	1.08 (0.79 to 1.47)	0.93 (0.63 to 1.37)
Depression	1.86 (1.17 to 2.96)	1.95 (1.13 to 3.39)	0.96 (0.64 to 1.44)	0.66 (0.39 to 1.11)
Anxiety	1.16 (0.63 to 2.13)	0.74 (0.35 to 1.53)	1.86 (1.19 to 2.90)	3.71 (2.06 to 6.87)
Diabetes	1.13 (0.67 to 1.91)	1.46 (0.80 to 2.65)	1.16 (0.77 to 1.75)	1.22 (0.74 to 2.01)
Osteoporosis	1.95 (0.99 to 3.85)	1.61 (0.75 to 3.43)	1.90 (1.08 to 3.32)	1.00 (0.53 to 1.90)
Cancer	2.13 (1.14 to 3.98)	1.64 (0.84 to 3.22)	2.54 (1.54 to 4.20)	2.52 (1.42 to 4.46)
Combined measures				
Cardiac disease*	1.26 (0.78 to 2.03)	1.22 (0.71 to 2.08)	2.08 (1.45 to 2.97)	1.68 (1.10 to 2.58)
Depression/anxiety†	1.44 (0.88 to 2.37)	1.44 (0.88 to 2.36)	1.37 (0.95 to 1.96)	1.82 (1.19 to 2.78)
Covariates				
Exacerbation in 2014	3.67 (2.40 to 5.63)	2.74 (1.71 to 4.39)	2.55 (1.79 to 3.62)	1.89 (1.25 to 2.87)
Sex, female	1.13 (0.76 to 1.68)	0.93 (0.59 to 1.46)	0.77 (0.57 to 1.05)	0.67 (0.46 to 0.99)
CAT-score in 2014	1.08 (1.05 to 1.10)	1.06 (1.03 to 1.09)	1.07 (1.05 to 1.09)	1.07 (1.04 to 1.09)
Education, high	1.20 (0.81 to 1.78)	1.39 (0.90 to 2.15)	0.86 (0.63 to 1.17)	1.02 (0.70 to 1.48)
Smoking				
Former smoker	0.79 (0.31 to 2.03)	0.91 (0.33 to 2.47)	1.44 (0.51 to 2.57)	1.40 (0.56 to 3.54)
Current smoker	0.87 (0.33 to 2.29)	0.93 (0.32 to 2.66)	1.28 (0.56 to 2.93)	1.71 (0.65 to 4.48)
Multilevel modelling				
Variance (95% CI)	0.08 (0.01 to 0.73)			
Intraclass correlation coefficient	0.024			

**Figure 2:**
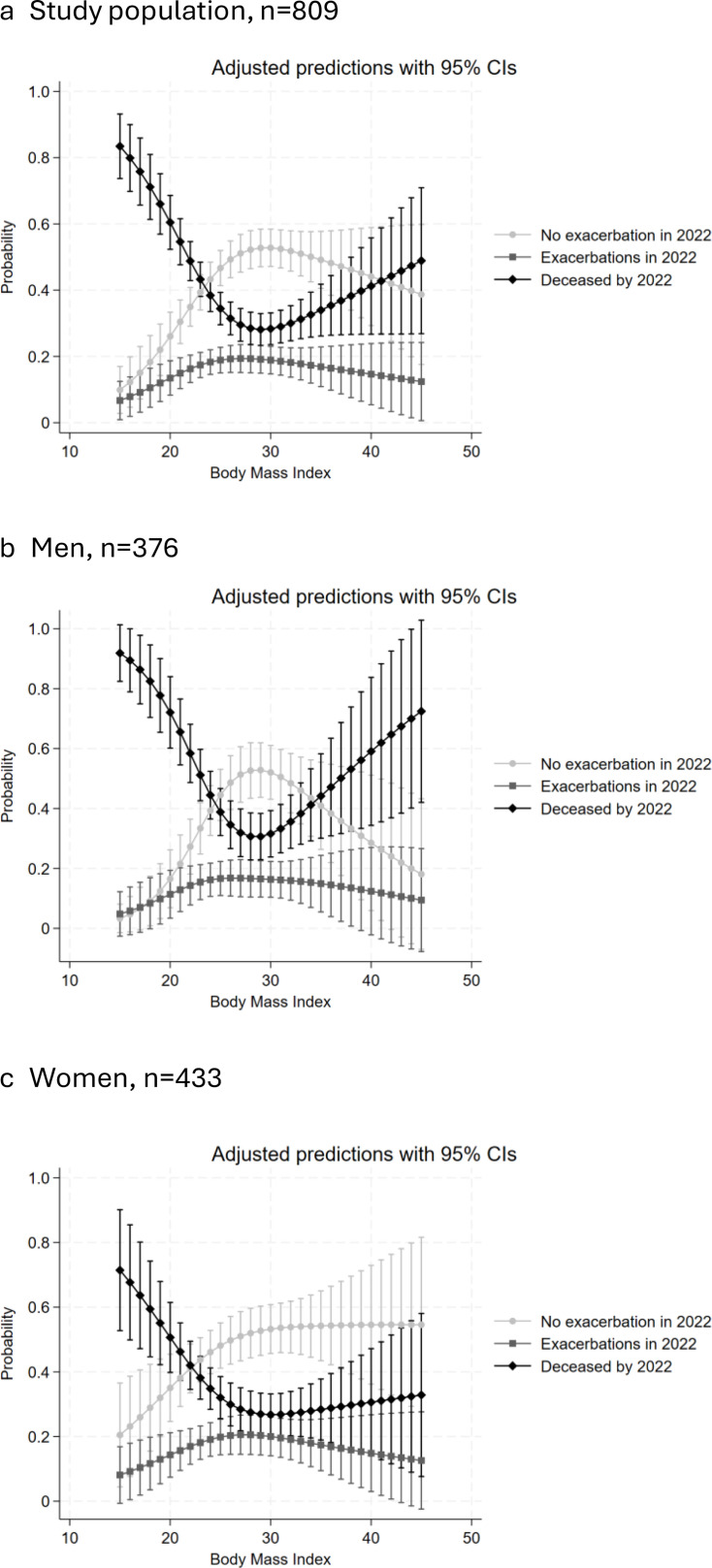
Predicted probabilities with 95% CIs for outcomes by body mass index with the other variables fixed at their means (fully adjusted model) in (a) the whole study population, n=809, (**b**) men, n=376 and (c) women, n=433. The probabilities for the three outcomes sum up to 1.0.

Multilevel multinomial regression analysis with no exacerbation in 2022 as reference category. The adjusted RRRs were obtained by controlling for age, age2 (results not shown), smoking, attained level of education, BMI with restricted cubic splines (results presented in figure 2), sex, CAT score and exacerbations in 2014.

* Model using a combined variable for chronic heart failure, ischaemic heart disease and atrial fibrillation.

† Model using a combined variable for depression and anxiety.

BMI, body mass index; CAT, COPD assessment test; COPD, chronic obstructive pulmonary disease; RRR, relative risk ratio; TIA, transient ischaemic attack.

For patients with COPD and a history of anxiety or cancer, we found statistically significantly increased risk ratios for mortality, compared with having no recent exacerbations. When stratified by sex, women had an RRR of 3.31 (95% CI 1.64 to 6.68) and men 4.62 (95% CI 1.42 to 15.09) for mortality associated with anxiety in 2014, but interaction testing did not indicate statistically significant effect modification by sex. The presence of cardiac disease in 2014, especially chronic heart failure, was associated with increased mortality risk, RRR 2.69 (95% CI 1.36 to 5.33). In unadjusted analyses, there were increased risks for all cardiac diseases, but after adjustment, it remained statistically significant only for chronic heart failure. We also found a raised risk of mortality for those who had exacerbations in 2014. There were no statistically significant associations with mortality for smoking or education.

The risk of mortality was found to be increased for both lower and higher values of BMI (figure 2a). For exacerbations, there was a slightly decreased risk with lower BMI. When stratified by sex, the U-shaped association with mortality was more evident for men than women, as the increased risk of mortality at higher BMI was less prominent among women (figure 2b,c).

### Sensitivity analyses 

The first sensitivity analysis found that results were consistent when the sample with complete data for covariates was compared with all available participants (data not shown). Second, multilevel logistic regression (for the surviving subpopulation) produced similar results for exacerbations in 2022 as the multilevel multinomial regression, although the risk estimates were of slightly, but not significantly, higher magnitude for positive associations, and of slightly lower magnitude for inverse associations (table 3). Also, the risk of exacerbations in 2022 increased with declining BMI for this population (online supplemental figure 1). Third, the results were consistent after removing the five centres with <5 participants.

**Table 3:** Multilevel analysis of COPD outcome in patients surviving to 2022

N=495	Exacerbations in 2022	
	Unadjusted	Adjusted
	OR (95% CI)	OR (95% CI)
Comorbidity		
Chronic heart failure	1.30 (0.57 to 2.93)	0.80 (0.29 to 2.24)
Ischaemic heart disease	1.60 (0.90 to 2.84)	1.62 (0.84 to 3.12)
Atrial fibrillation	1.62 (0.75 to 3.50)	2.46 (0.97 to 6.29)
Stroke/TIA	0.81 (0.36 to 1.80)	0.72 (0.29 to 1.77)
Hypertension	0.83 (0.55 to 1.23)	0.79 (0.48 to 1.30)
Depression	1.89 (1.17 to 3.04)	2.24 (1.21 to 4.15)
Anxiety	1.15 (0.62 to 2.16)	0.63 (0.28 to 1.44)
Diabetes	1.13 (0.66 to 1.93)	1.42 (0.80 to 2.65)
Osteoporosis	2.04 (1.01 to 4.11)	1.57 (0.69 to 3.55)
Cancer	2.18 (1.14 to 4.14)	1.39 (0.66 to 2.92)
Combined measures		
Cardiac disease*	1.25 (0.77 to 2.04)	1.36 (0.77 to 2.39)
Depression/anxiety†	1.53 (0.97 to 2.42)	1.49 (0.88 to 2.54)
Covariates		
Exacerbation in 2014	3.76 (2.42 to 5.85)	2.92 (1.78 to 4.77)
Sex, female	1.12 (0.75 to 1.68)	0.99 (0.61 to 1.61)
CAT-score in 2014	1.08 (1.05 to 1.11)	1.07 (1.03 to 1.10)
Education, high	1.20 (0.80 to 1.79)	1.45 (0.92 to 2.31)
Smoking		
Former smoker	0.79 (0.30 to 2.05)	1.00 (0.34 to 2.94)
Current smoker	0.87 (0.33 to 2.33)	0.96 (0.31 to 2.98)
Multilevel modelling		
Variance (95% CI)	0.14 (0.01 to 1.76)	
Intraclass correlation coefficient	0.041	

Multilevel logistic regression analysis with no exacerbation in 2022 as reference category. The adjusted ORs were obtained by controlling for age, age2 (results not shown), smoking, attained level of education, BMI with restricted cubic splines (results presented in online supplemental figure 1), sex, CAT score and exacerbations in 2014.

* Model using a combined variable for chronic heart failure, ischaemic heart disease and atrial fibrillation.

† Model using a combined variable for depression and anxiety.

BMI, body mass index; CAT, COPD assessment test; COPD, chronic obstructive pulmonary disease; TIA, transient ischaemic attack.

## Discussion

The main finding of this cohort study with an 8-year follow-up was that the variance between healthcare centres for COPD outcomes was small and individual patient factors appeared to be of greater importance. Patients with COPD and a history of depression had a doubled risk of new exacerbations, and those with a history of anxiety had an almost four times increased risk of mortality. The selection effect of mortality in a panel study may affect the magnitude of some associations, and its influence on results must be considered carefully.

Our study included patients from 70 primary care centres with differences in size, location, staffing and proximity to secondary care. The majority had nurse-led COPD clinics and local or regional guidelines for management of COPD. Despite this, the estimated variability in COPD outcomes between centres was small. A possible interpretation is that management of patients with COPD in primary care in Sweden is rather uniform and standardised among the centres included in this study. A previous Swedish study reported that structured management of patients with COPD in primary care was increasing during 1999–2009, and patients who attended healthcare centres with nurse-led COPD clinics had fewer exacerbations and hospital admissions.^14^ However, our finding of small variability between centres aligns with previous studies of low variability in primary care. A Canadian study reported variability of 1%–3% between primary care clinics for recognition and treatment of depression.^15^ A Spanish study of variability in health outcomes and costs found that less than 3% of the prescriptions, 5% of referrals and 9% of visits to primary care physicians were attributed to the differences between healthcare centres.^9^ Although these earlier studies are from different countries and diagnoses, the results seem consistent and support the finding that outcomes are explained more comprehensively by individual patient factors. Our use of multilevel modelling in this context can provide more effective adjustment for centre characteristics, reducing the risk of residual confounding, while improving accuracy by allowing estimates of associations between patient characteristics and outcomes to vary by centre.

Comorbid depression has been associated with increased risk of both exacerbations and mortality in patients with COPD.^3 16 17^ Anxiety has been identified as one of twelve diseases predictive of mortality in patients with COPD, especially in women.^5^ In some studies, anxiety has been associated with increased risk of exacerbations, while others have not shown an association.^3 17 18^ We found that depression was associated with an increased risk of recent exacerbations in the patients surviving to 2022, but there was no association with mortality. Our study indicated an increased risk of mortality in both men and women associated with anxiety. Anxiety is common in patients with multimorbidity, and the high magnitude risk estimate we found might reflect anxiety as an independent risk factor or as a marker of chronic disease severity and worse health status.^19^ Anxiety and depression often co-occur in patients with COPD.^20^ When we repeated the analysis with anxiety and depression combined as one variable, we found increased risks for both mortality and future exacerbations, although statistically significant results were only seen for mortality. A potential reason for the somewhat divergent results may be that anxiety and depression diagnoses were identified in the medical record review, so we have no knowledge of the diagnostic criteria, or the duration of symptoms. Similarly, we could only identify exacerbations during the previous 6 months through the questionnaire 2022, thereby likely only identifying surviving participants with more frequent exacerbations. We have no knowledge of exacerbations occurring between 2014 and 2022, and participants who died before 2022 are likely to have had unreported exacerbations prior to their death. However, by modelling both exacerbations and mortality as outcomes in the same model, it was possible to assess markers of adverse outcomes more comprehensively than if those who died were simply excluded.

Comorbid cardiovascular disease has been associated with increased mortality, and chronic heart failure as well with an increased risk of exacerbations.^3 21^ In our study, we found increased risks for mortality associated with all cardiac diseases in the univariate analyses. The risk remained statistically significant only for heart failure and the combined cardiac disease variable in the adjusted models, due to multicollinearity, as patients often have more than one cardiac diagnosis: the combined cardiac disease variable may give an indication of risk magnitude that is more readily interpretable.

Our finding that a history of exacerbations is associated with both increased risk of future exacerbations and mortality is consistent with previous studies, including those that used spirometry.^3 22^ Even though we were unable to distinguish between severe and moderate exacerbation episodes in our study, the associations were statistically significant. The most recent years of our follow-up period were during the COVID-19 pandemic, and this may have influenced the results. During the pandemic, there was an increase in prescriptions of steroids and antibiotics used for COPD exacerbations, and this prescription pattern may have been reflected in the information collected in 2022.^23^ Other exposures that have changed over time include the prevalence of smoking. Prior studies have reported heterogeneous results for the association of smoking with exacerbations, and we found no association between smoking and risk of exacerbations.^24 25^

For BMI, we observed a U-shaped association with mortality, as reported in a recent meta-analysis.^6^ For exacerbations, we did not find any difference in risk between normal and high BMI, consistent with previous research.^7^ However, an increased risk of exacerbations in underweight patients has been reported,^7^ which we did not find in the multinomial logistic regression where mortality was one of the outcome categories. When analysing the survivors separately, without having mortality as a competing risk, we did find an increased risk of exacerbations associated with lower BMI. Thus, the patients with low BMI in 2014, who survived until 2022, appear to have an increased risk of exacerbations that was not seen in the entire sample as low BMI was a greater risk for mortality.

The clinical relevance of our findings is the importance of individualised care of patients with COPD in primary care, as in a uniform primary care system, individual patient factors such as comorbidity and BMI may be the main drivers of adverse outcomes. This includes identifying and treating mental health conditions (including identifying somatic risks that underlie these conditions) and closer monitoring of patients with heart failure or previous exacerbations could help prevent poor outcomes.

### Strengths and limitations

This study had more than 800 randomly selected patients with COPD from primary care with an 8-year follow-up. The use of both medical record reviews and questionnaires for data collection enabled us to use the combination of prospectively recorded diagnoses with the patients’ own experience of their health. Another strength was the use of multilevel multinomial logistic regression for the analysis. Multilevel modelling accounts for differences between healthcare centres more effectively than some other methods, and multinomial logistic regression allowed mortality to be modelled as a competing outcome. Considering the high mortality rate at follow-up (31%), it was important to account for the role of mortality in shaping the results. A sensitivity analysis, including only the survivors, found similar but subtly different results for associations with exacerbations in 2022 for comorbidity and previous exacerbations. The choice of study population at follow-up in a panel study may thus influence the magnitude of the results, although the direction of associations remained similar in this study. This emphasises the importance of considering the influence of mortality in a panel study, as selection effects may alter the magnitude of some associations.

There are some potential limitations. The non-response rate in 2014 was 41%, and 32% at follow-up in 2022. The respondents in 2014 were somewhat older than those who did not respond, and the respondents in 2022 may have had a slightly better COPD-specific health status in 2014 (14 compared with 15), although the suggested minimal clinically important difference for CAT is two units.^26^ Non-response may be a cause of potential selection bias and may also limit variability between centres. The loss of patients in the main analysis due to missing data for covariates was 11%. To examine the consequences, we performed a sensitivity analysis, with consistent results. Data on exacerbations were obtained from the questionnaires, and to reduce the risk of recall bias, a period of 6 months was used. Hence, we lack knowledge of exacerbations occurring for most of the time between 2014 and 2022, and participants with frequent exacerbations during this time may have died prior to 2022. Thus, the estimates of risk of exacerbation should only be understood as the risk of exacerbation in 2022, among patients who were still alive. Similarly, we have no knowledge of comorbid conditions that developed during the follow-up, or treatment compliance. Height and weight were self-reported, but BMI based on self-reported measures has been found to have acceptable agreement with measured BMI in Sweden.^27^

Some participants may have changed healthcare centres during follow-up, possibly making the estimation of variability among the healthcare centres less precise. Finally, while healthcare centres in more disadvantaged areas of Sweden may have been under-represented, our study population likely broadly represents Swedish patients with a diagnosis of COPD. Patients may have had respiratory symptoms for a long time before being diagnosed with COPD, but having comorbidity might lead to an earlier diagnosis.^28^ Thus, there is a possibility of selection bias: individuals with other pre-existing chronic diseases are more likely to seek care and receive a COPD diagnosis.

## Conclusions

The variability between healthcare centres for the likelihood of COPD exacerbations and mortality is small, indicating that individual patient factors may be of more importance for COPD outcomes than differences in primary care settings. It is of note that a history of anxiety was associated with future excess mortality risk, and this finding should be investigated further for replication. As expected, pre-existing cardiovascular disease, particularly heart failure, is associated with increased mortality risk in patients with COPD.

## Supplementary material

10.1136/fmch-2025-003713 online supplemental file 1

## Data Availability

Data are available on reasonable request.
