# Supplementary material for: Risk factors for COPD exacerbations and mortality, and variation between primary care settings: the PRAXIS cohort study in Sweden
Source: Fam Med Community Health. 2026 Feb 19;14(1):e003713. doi: 10.1136/fmch-2025-003713 (PMC12927360; doi:10.1136/fmch-2025-003713)

Supplementary figure 1 Predicted probability with 95% confidence intervals (CI) for exacerbations in 2022 by body mass index (BMI) with the other variables fixed at their means. From logistic regression with no exacerbation in 2022 as reference category. N=495.

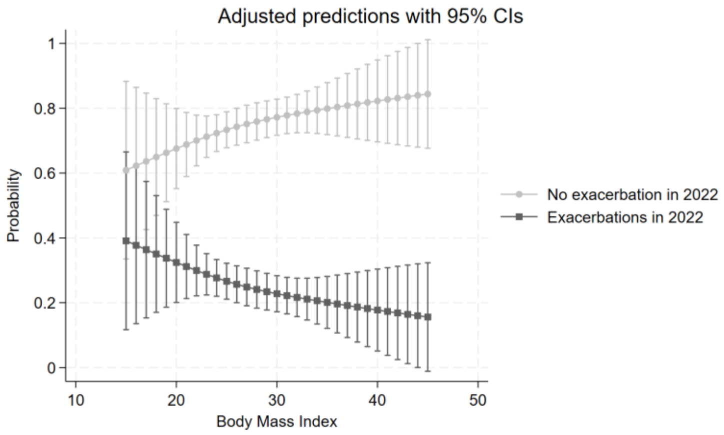

Supplement: online supplemental file 1 [file fmch-14-1-s001.pdf]
